# Prevalence of diabetic nephropathy in the diabetes mellitus population: A protocol for systematic review and meta-analysis

**DOI:** 10.1097/MD.0000000000031232

**Published:** 2022-10-21

**Authors:** Sicheng Li, Huidi Xie, Yang Shi, Hongfang Liu

**Affiliations:** a Nephrology Department, Dongzhimen Hospital Affiliated to Beijing University of Chinese Medicine, Beijing, China.

**Keywords:** diabetes mellitus, diabetic nephropathy, meta-analysis, prevalence, protocol, systematic review

## Abstract

Diabetic nephropathy (DN) is the leading cause of end-stage renal disease worldwide, placing enormous pressure on healthcare systems and creating a heavy socioeconomic burden. It is urgent to comprehensively study the epidemiological characteristics of DN in diabetic patients and to analyze the related factors to its incidence in order to implement effective prevention and control measures.

**Methods and analysis:**

Computer-aided searches of the MEDLINE, EMBASE, Web of Science, PsycINFO, and CINAHL databases will be performed for prospective cohort studies reporting the prevalence of DN in diabetic populations. Studies will be pooled using a generalized linear mixed model, and a single proportion of included studies will be calculated to derive the overall incidence of DN in the diabetic population, and to analyze the effect of different factors on the incidence of DN. Publication bias will be assessed using a funnel plot combined with Begg test. Sensitivity analyses will be performed using the separation method, the exclusion of low-quality studies, and the trim and fill method.

**Results:**

The primary outcome will be the prevalence of DN in the diabetic population; secondary outcomes will be the influence of factors such as age, gender, region, ethnicity, duration of diabetes, type of diabetes, baseline body mass index, baseline glycated hemoglobin level, baseline blood pressure, quality of included studies, and follow-up time on the prevalence of DN in diabetic patients.

**Conclusion:**

Through this systematic review and meta-analysis, the study will more comprehensively obtain the prevalence of DN in diabetic populations worldwide, and gain a deeper understanding of the differences in the prevalence of DN in diabetic populations with different characteristics, so as to provide evidence for the management of diabetes and the prevention of DN.

## 1. Introduction

Diabetes mellitus is a major public health problem worldwide.^[1]^ Epidemiological studies show that the prevalence of diabetes in adults was about 8.8% in 2015, and this figure will rise to 10.4% by 2024. It is estimated that the number of patients with diabetes and diabetic nephropathy (DN) in China has reached 24.3 million.^[2,3]^ DN, which refers to chronic kidney disease caused by diabetes, is one of the major microvascular complications of diabetes and the leading cause of death in diabetic patients.^[4]^ Although our current understanding of the disease process has improved, unlike other diabetes complications, the prevalence of DN has not declined over the past few decades.^[5]^

DN is characterized by microalbuminuria, overt proteinuria, and decreased glomerular filtration rate as the main clinical manifestations, and can eventually develop into end-stage renal disease.^[6]^ The disease is pathologically characterized by mesangial expansion and hypertrophy, extracellular matrix protein deposition, and podocyte apoptosis, which can cause glomerulosclerosis and tubulointerstitial fibrosis, ultimately leading to renal failure.^[7]^ DN is a multifactorial disease. Many pathophysiological processes, including inflammation, oxidative stress, and renal hemodynamic changes, are involved in the pathogenesis of DN. These factors may have a strong impact on the occurrence and progression of DN.^[8,9]^ Given the severe health consequences of DN, the enormous pressure on the healthcare system, and the heavy socioeconomic burden,^[6,10,11]^ a comprehensive assessment of the epidemiological characteristics and risk factors of DN in diabetic patients to implement effective prevention and control measures is very urgent and necessary.

To date, a large number of epidemiological studies on the risk factors of DN have been carried out worldwide. However, a search of the Web of Science, Embase and Medline databases and the international prospective register of systematic reviews (PROSPERO) found that only three systematic reviews were relevant to the topic “prevalence of kidney disease in diabetic patients”, and there were strict geographic restrictions on included studies and a lack of in-depth analysis of factors associated with the pathogenesis of DN.^[12,13]^ Due to the high degree of heterogeneity in the pathogenesis of DN, the existing evidence is far from thorough and comprehensive.^[14,15]^ Therefore, we will conduct a comprehensive qualitative and quantitative analysis of prospective cohort studies investigating the prevalence of DN in diabetes mellitus patients to improve our understanding of the factors associated with prevalence and occurrence of DN.

## 2. Methods

### 2.1. Study registration

This study followed the Meta-analysis of Observational Studies in Epidemiology guidelines and preferred reporting items for systematic reviews and meta-analyses (the PRISMA statement), and the study protocol was registered with International Prospective Register of Systematic Reviews on May 10, 2022, with registration number: CRD42022320003.

### 2.2. Ethics and dissemination

Ethical approval was not required for this study because this study was a reanalysis of the original study and did not address participant privacy concerns. The results of this study will provide information on the prevalence of diabetes and associated risk factors in people with diabetes, and the protocol will be disseminated by a peer-reviewed journal, providing relevant evidence for the primary and secondary prevention of DN.

### 2.3. Inclusion and exclusion criteria

#### 2.3.1. Participants.

Individuals diagnosed with diabetes will be included regardless of age, gender, ethnicity, and region. We will include studies that specify valid diabetes diagnostic criteria at the start of the study, including but not limited to those provided by the American Diabetes Association,^[16–18]^ those provided by the WHO,^[19]^ and others recognized diagnostic criteria for diabetes.^[20]^

#### 2.3.2. Exposure and control.

It will not be involved in this study. This study is aimed to comprehensively investigate the prevalence of DN and its associated risk factors in diabetic patients, without involving any other exposure and control factors.

#### 2.3.3. Types of studies.

This study will include all prospective cohort studies published in English reporting the point prevalence of DN in diabetic patients. A study will be excluded if it could not provide detailed data on the point prevalence of DN in people with diabetes. In addition, historical cohort studies will not be within the scope of this study.

#### 2.3.4. Outcomes.

##### 2.3.4.1. Primary outcomes.

Prevalence of DN: The diagnosis of DN should be based on recognized diagnostic criteria, such as ADA 2008,^[16]^ ADA 2010,^[17]^ and KDOQI 2007.^[21]^

##### 2.3.4.2. Secondary outcomes.

Factors associated with the onset of DN: this study will specifically analyze the impact of the factors of age, gender, region, ethnicity, duration of diabetes, type of diabetes, baseline body mass index (BMI), baseline glycated hemoglobin level, baseline blood pressure, quality of included studies, follow-up time on the prevalence of DN in diabetic patients.

### 2.4. Retrieval strategy

MEDLINE, EMBASE, Web of Science, PsycINFO, and Cumulative Index to Nursing and Allied Health Literature databases will be searched, using subject headings combined with free words. The retrieval strategy of MEDLINE is shown in Table 1. In other databases, the retrieval strategy will be adjusted according to the characteristics of the database. To be clear, we also plan to manually search the references of relevant reviews and systematic reviews to further identify additional potentially eligible studies.

**Table 1:** Search strategy of Ovid MEDLINE(R) ALL.

1	exp Diabetes Mellitus/
2	(diabetic* or IDDM or NIDDM or MODY or T1DM or T2DM).tw.
3	1 or 2
4	exp Diabetic Nephropathies/
5	diabetic nephropath$.tw.
6	((diabetic or diabetes) and (kidney$ or renal or nephro$ or nephritis or glomerulo$)).tw.
7	4 or 5 or 6
8	cohort studies/ or follow-up studies/ or longitudinal studies/ or prospective studies/
9	(Incidence or Prevalence or epidemiolog* or cohort or Prospective).tw.
10	8 or 9
11	English.lg.
12	3 and 7 and 10 and 11
13	Animals/
14	Humans/
15	13 and 14
16	13 not 15
17	12 not 16

### 2.5. Data collection and management

#### 2.5.1. Study screening.

**Figure 1.**
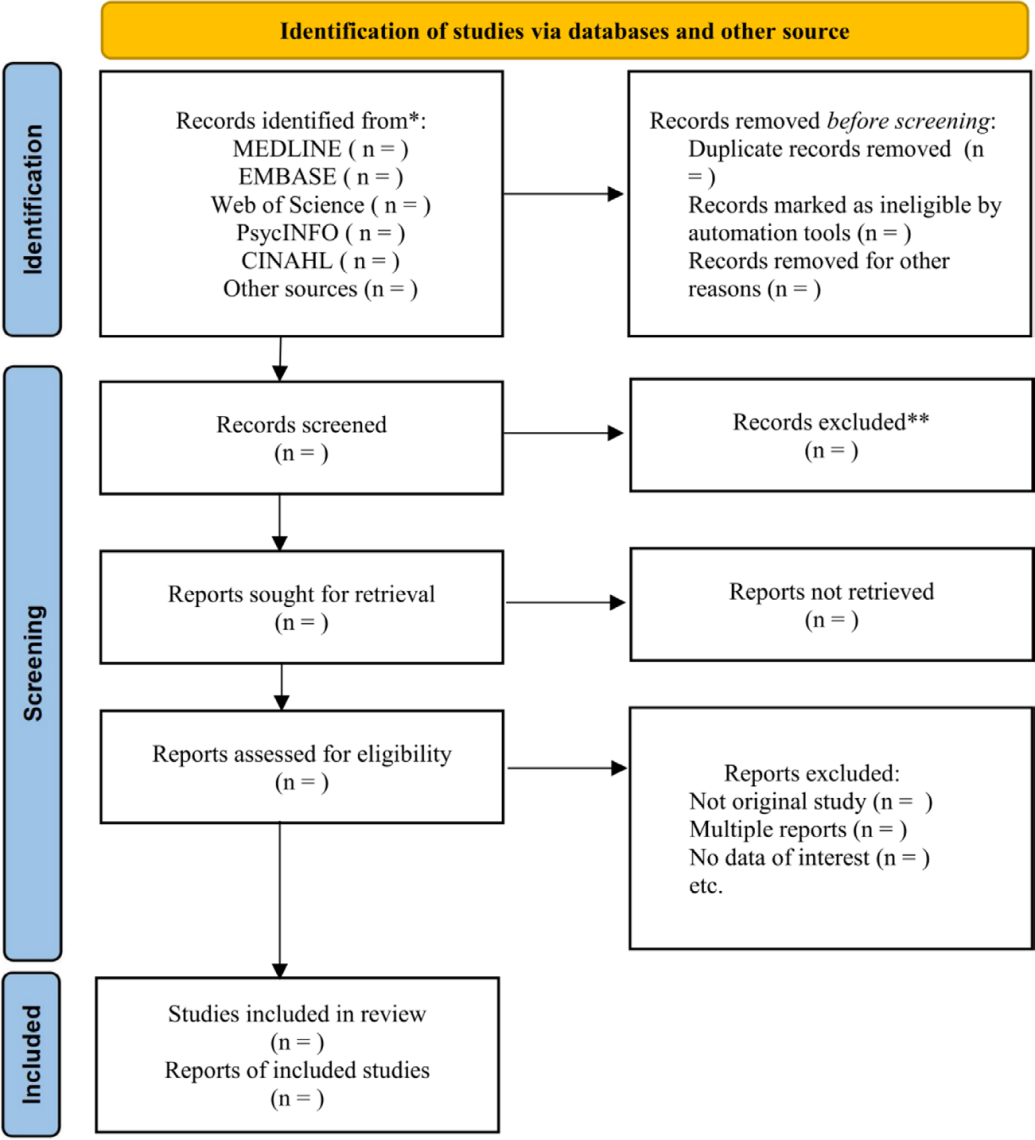
PRISMA flow diagram. PRISMA = preferred reporting items for systematic reviews and meta-analyses.

The retrieved results will be imported into Endnote X7 and duplicate records will be removed by Endnote X7. Results will be initially screened by 2 investigators (HX and YS) by independently reading titles and abstracts, and the full texts of these potentially eligible studies will be independently read by 2 investigators to determine whether the records meet the inclusion criteria. Any disagreement between the screening results of the two investigators will be resolved by discussion and, if necessary, will be further evaluated by a third investigator (SL) until agreement is reached. The PRISMA flow diagram is presented in Figure 1.

#### 2.5.2. Data extraction.

Data from eligible studies will use a pre-designed data extraction table, and the extracted information will be managed using Excel, including study population, age, gender, ethnicity, region where the study was conducted, duration of diabetes, type of diabetes, baseline (BMI), baseline glycated hemoglobin level, baseline blood pressure, quality of included studies, duration of follow-up, etc. Data extraction will be performed independently by two investigators (HX or YS). Disagreement in results were resolved by discussion and, if necessary, further evaluation by a third investigator (HL) until agreement is reached.

#### 2.5.3. Evaluation of research quality.

As recommended by the Chinese Cochrane Centre, the Newcastle-Ottawa Quality Assessment Scale will be used for quality assessment of eligible prospective cohort studies.^[22]^ The process of quality assessment will be done according to the Newcastle-Ottawa Quality Assessment Scale coding manual and the assessment of the quality of the included studies will be done by considering the following characteristics:

##### 2.5.3.1. Selection category.

(1) Representativeness of the exposed cohort
(2) Selection of the non-exposed cohort
(3) Ascertainment of exposure
(4) Demonstration that outcome of interest was not present at start of study

##### 2.5.3.2. Comparability category.

(1) Comparability of cohorts on the basis of the design or analysis

##### 2.5.3.3. Exposure category.

(1) Assessment of outcome
(2) Was follow-up long enough for outcomes to occur
(3) Adequacy of follow up of cohorts

#### 2.5.4. Handling of missing data.

If the research report does not provide all the data in the data extraction form, we will attempt to contact the corresponding author or the first author to obtain the data by email. If no feedback is received, only the available data will be analyzed.

### 2.6. Analysis

#### 2.6.1. Data synthesis and heterogeneity assessment.

Studies will be pooled using a generalized linear mixed model, and a single proportion of included studies will be calculated^[23]^ to calculate the overall incidence of DN in the diabetic population.

Heterogeneity between studies will be assessed by calculating the standard Cochran *Q* test and *I*^2^ statistic, and all statistical analyses will be performed by R software. *I*^2^ values of 25% or below, nearly 50%, and above 75%, respectively, with *P* < .05, can be considered as low, moderate, and high heterogeneity accordingly. When studies are found to be statistically heterogeneous, random-effects models will be used to calculate the prevalence of DN with 95% confidence intervals and pooled data. Otherwise, a fixed effects model will be applied.

#### 2.6.2. Meta regression.

If the number of included studies is greater than or equal to 10, then we will perform meta regression based on methodological characteristics and demographic characteristic of study population, age, gender, ethnicity, region in which the study was conducted, duration of diabetes, type of diabetes, BMI at baseline, baseline glycated hemoglobin level, baseline blood pressure, quality of included study, follow-up time. The *P* value of meta regression is used to evaluate the differences in the prevalence of DN among diabetic patients with different characteristics.

#### 2.6.3. Subgroup analysis.

If there is heterogeneity among multiple studies, we will conduct a targeted subgroup analysis based on the demerits of meta-regression to deal with the heterogeneity, analyze the source of the heterogeneity, and clarify the prevalence of DN in the diabetic population with different characteristics.

#### 2.6.4. Publication bias.

The visual symmetry of the funnel plot will be used to assess potential publication bias. If the number of included studies is greater than or equal to 10, we will conduct a further assessment of publication bias by Begg test. If *P* < .05, it will be considered to have significant publication bias.

#### 2.6.5. Sensitivity analysis.

Leave-one-out method sensitivity analysis was used to investigate the effect of individual studies on the pooled results. The low-quality studies (0–4 points) are proposed to see whether the quality of the studies affected the results of the studies. If there is significant publication bias, a trim and fill method sensitivity analysis will be performed.

## 3. Discussion

DN caused by diabetes is one of the main causes of end-stage renal failure and has become an urgent public health problem worldwide.^[24]^ The global prevalence of diabetes is increasing rapidly, especially in developing countries.^[25,26]^ As the prevalence of diabetes increases, the prevalence of DN is also expected to increase if clinical strategies to prevent DN do not improve immediately. The development and progression of DN are related to many factors.^[27]^ It is very important to identify the prevalence and related factors of DN and to formulate targeted measures for its prevention and control.^[28,29]^ The prevalence of DN and the related factors affecting its onset are poorly understood. The purpose of this systematic review is to comprehensively assess the prevalence of DN in the diabetic population worldwide, and to analyze the differences in the prevalence of DN in the diabetic population with different characteristics, so as to prospectively analyze the related factors of its incidence, and provide evidence for the management of diabetes mellitus and the prevention of DN.

## Author contributions

**Conceptualization:** Sicheng Li.

**Data curation:** Huidi Xie and Yang Shi.

**Formal analysis:** Sicheng Li.

**Funding acquisition:** Hongfang Liu.

**Investigation:** Hongfang Liu.

**Methodology:** Sicheng Li.

**Project administration:** Sicheng Li.

**Software:** Huidi Xie and Yang Shi.

**Writing – original draft:** Sicheng Li.

**Writing – review & editing:** Hongfang Liu.
